# Bioguided Isolation of Antibiofilm and Antibacterial Pregnane Glycosides from *Caralluma quadrangula*: Disarming Multidrug-Resistant Pathogens

**DOI:** 10.3390/antibiotics10070811

**Published:** 2021-07-03

**Authors:** Riham A. El-Shiekh, Mariam Hassan, Rasha A. Hashem, Essam Abdel-Sattar

**Affiliations:** 1 Department of Pharmacognosy, Faculty of Pharmacy, Cairo University, El-Kasr El-Aini Street, Cairo 11562, Egypt; riham.adel@pharma.cu.edu.eg; 2 Department of Microbiology and Immunology, Faculty of Pharmacy, Cairo University, El-Kasr El-Aini Street, Cairo 11562, Egypt; rasha.hashem@pharma.cu.edu.eg

**Keywords:** antivirulence, *Acinetobacter baumannii*, *Caralluma quadrangula*, in vivo, methicillin-resistant *Staphylococcus aureus* (MRSA), pregnane glycosides, skin infection

## Abstract

Methicillin-resistant *Staphylococcus aureus* (MRSA) and multidrug-resistant *Acinetobacter baumannii* (MDRAB) present a serious challenge because of their capability to cause biofilm resistance to commonly used antibiotics producing chronic infections and hindering the process of wound healing. In the current study, we investigated the antibacterial activity of *Caralluma quadrangula* extracts (MeOH, and its fractions CH_2_Cl_2_ and *n*-butanol) against multidrug-resistant MRSA USA300 and *A. baumannii* AB5057. In vitro, the MeOH extract and both fractions of *C. quadrangula* significantly inhibited biofilm formation and disrupted previously established biofilm by MRSA and MDRAB at all the tested concentrations (0.625, 0.313, and 0.156 mg/mL). In vivo, *C. quadrangula* extracts successfully decreased bacterial loads in MRSA-infected skin lesions in mice. Four pregnane glycosides and one flavone glycoside were isolated from the bioactive *n*-butanol fraction. The isolated compounds (**Rus A**–**E**) were tested for their biofilm inhibition and biofilm detachment activities. The results revealed that **Rus C** was the most active compound (IC_50_ = 0.139 mmole), while **Rus E** was the least active (IC_50_ = 0.818 mmole). These results support the potential use of *C. quadrangula* extracts or their isolated compounds for hindering the biofilm attachment and the virulence of MRSA and MDRAB and their application as a topical antimicrobial preparation for MRSA skin infections.

## 1. Introduction

Methicillin-resistant *Staphylococcus aureus* (MRSA) and multidrug-resistant *Acinetobacter baumannii* (MDRAB) are opportunistic pathogens. They are well fitted to hospital environments, and the perseverance of their chronic infections is mainly due to their capability to form biofilm and resist the host immune system [1,2]. The emergence of microbial resistance to numerous conventional antibiotics has become a serious global concern. These microbes cause serious bloodstream infections, urinary tract infections, wound infections, pneumonia, and secondary meningitis with high morbidity and mortality rates [3,4,5]. MRSA and MDRAB are the most commonly encountered pathogens in hospitals [6,7]. Drug resistance and biofilm formation appear to play a critical condition in the pathogenicity of MRSA and MDRAB [1,2,8]. The majority of *A. baumannii* were found to be multidrug-resistant strains. Carbapenem-resistant *A. baumannii* was reported among the predominant isolates that emerged in healthcare systems [9]. Recently, colistin-resistant strains have been isolated from wounds and hospital-resident patients, which is considered as the last line of defense against multidrug-resistant strains [10,11]. Hence, screening new naturally occurring candidates for fighting these microbes is seriously needed, as these natural resources represent rich reservoirs for new antimicrobials and bioactive metabolites [12,13,14].

Several members of the genus *Caralluma* are succulent perennial edible plants with several traditional uses such as antiinflammatory, antidiabetic, antirheumatic, antiulcer, and wound healing [15,16]. Several *Caralluma* species or their phytoconstituents showed a wide spectrum of antimicrobial activity against fungi, Gram-positive and Gram-negative [17,18,19,20,21]. The major bioactive molecules identified in the members of the genus *Caralluma* are pregnane glycosides, flavone glycosides, terpenoids, and sterols showing a lot of therapeutic significance. *Caralluma quadrangula* (Forssk.) is a leafless succulent herb traditionally used for the treatment of ulcers, diabetes, and rheumatic arthritis [15,16]. Extract of *C. quadrangula* was applied in Saudi folk medicine in the treatment of freckles, melasma, diabetes, and vitiligo and in cases of hunger and thirst [22]. Abdel-Sattar and coworkers [23] reported a promising hypoglycemic effect for the methanolic extract of *C. quadrangula* (200 mg/kg) and its related fractions (dichloromethane and *n*-butanol fractions at a dose of 100 mg/kg), as well as russelioside B, the major isolated pregnane glycoside. In addition, a major isolated pregnane glycoside, russelioside B, showed several biological activities viz. antidiabetic [24], antiobesity [25], antiarthritic agent [26], and antiulcer [27]. Several *Caralluma* species showed a wide spectrum of antibacterial and/or antifungal activity [20,21].

Therefore, we investigated the antibiofilm and antibacterial activity of *C. quadrangula* extracts opposed to MRSA USA300 and MDRAB AB5057 in vitro. In addition, we accessed their efficacy in MRSA skin infection lesions in vivo.

## 2. Results

### 2.1. Minimum Bactericidal Concentration (MBC) and Antibiofilm Assays for C. quadrangula Extracts

**Figure 1:**
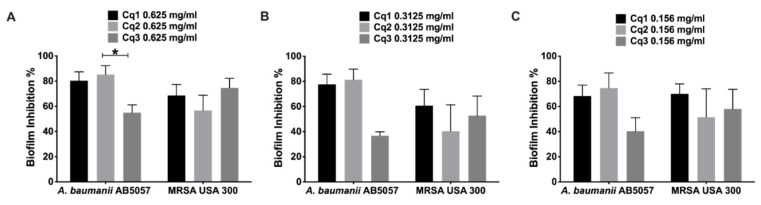
Biofilm inhibition activity. Effect of different concentrations (**A:** 0.625 mg/mL, **B:** 0.3125 mg/mL, and **C:** 0.156 mg/mL) of *Caralluma quadrangula* extracts (Cq1, Cq2, and Cq3) against MDRAB AB5057 and MRSA USA300 biofilm formation. Results are expressed as mean biofilm inhibition % ± standard error. * Indicates that the difference is significant at *p* < 0.05 (two-way ANOVA, Tukey’s post-hoc test).

*C. quadrangula* extracts (Cq1: methanolic extract, Cq2: methylene chloride, and Cq3: *n*-butanol fractions) were tested for in vitro antibacterial activity against MDRAB AB5057 and MRSA USA300. *C. quadrangula* extracts (Cq1, Cq2, and Cq3) showed antibacterial activity opposed to MDRAB AB5057 and MRSA USA300 with minimum bactericidal concentration (MBC) values of 12.5 mg/mL and 25 mg/mL, respectively. The three tested extracts inhibited MDRAB AB5057 and MRSA USA300 biofilm formation significantly at the tested concentrations (0.625, 0.313, and 0.156 mg/mL) (two-way ANOVA and Tukey’s test, *p* < 0.05) (Figure 1). There was no significant difference between the tested extracts in inhibitory activity against MDRAB AB5057 and MRSA USA300 towards biofilm formation at different tested concentrations except for Cq2 (0.625 mg/mL) and Cq3 (0.625 mg/mL) against MDRAB AB5057 (two-way ANOVA and Tukey’s test, *p* < 0.05, Figure 1).

**Figure 2:**
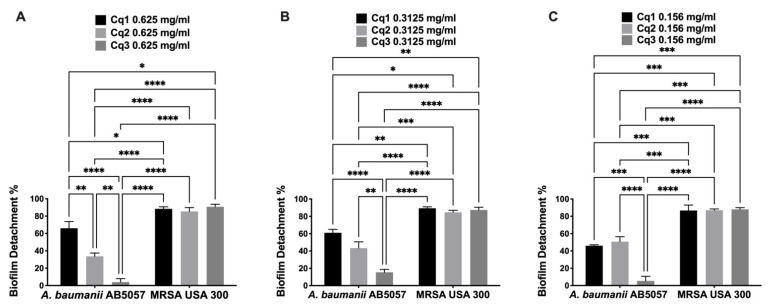
Biofilm detachment activity. Effect of different concentrations (**A:** 0.625 mg/mL, **B:** 0.3125 mg/mL, and **C:** 0.156 mg/mL) of *Caralluma quadrangula* extracts (Cq1, Cq2, and Cq3) on previously established MDRAB AB5057 and MRSA USA300 biofilm. Results are expressed as mean biofilm detachment % ± standard error. *, **, ***, and **** indicate that the difference is significant at *p* < 0.05, *p* < 0.005, *p* < 0.0005, and *p* < 0.0001, respectively (two-way ANOVA, Tukey’s post-hoc test).

Interestingly, the three tested extracts showed a significant biofilm detachment activity against MRSA USA300 stronger than against *A. baumannii* AB5057 at all the tested concentrations (0.625, 0.313, and 0.156 mg/mL) (two-way ANOVA and Tukey’s test, *p* < 0.05) (Figure 2). Regarding the biofilm inhibition activity, there was no significant difference between the tested extract concentrations (0.625, 0.313, and 0.156 mg/mL) against the two tested pathogens (MRSA USA300 and MDRAB AB5057) (two-way ANOVA, Tukey’s post-hoc test, *p* < 0.05). The same was observed regarding the biofilm detachment activity except for a significant difference between the activity of extract Cq1 at concentration 0.625 mg/mL and Cq1 at the least-tested concentration 0.156 mg/mL against MRSA USA300 (two-way ANOVA, Tukey’s post-hoc test, *p* < 0.05).

### 2.2. In Vivo Evaluation of C. quadrangula Extracts against MRSA USA300 Skin Infection

**Figure 3:**
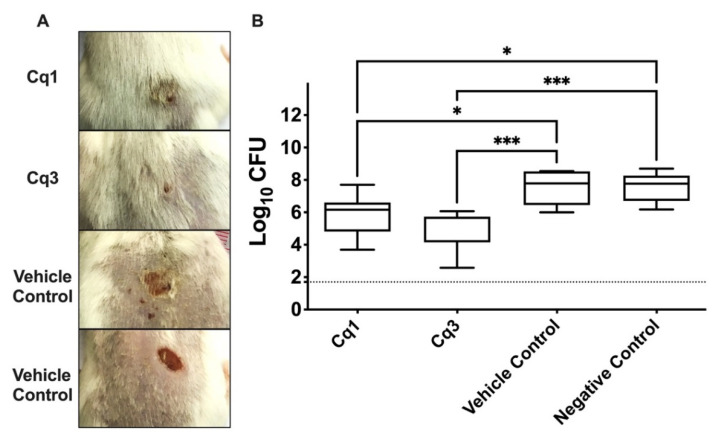
Efficacy of *Caralluma quadrangula* extracts (Cq1 and Cq3) in an in vivo murine model of MRSA skin infection. Thirty-two BALB/C mice were divided into four groups (n = 8), three treated groups (Cq1, Cq3, and vehicle), and the fourth group remained untreated as the negative control. (**A**) Efficacy of *Caralluma quadrangula* extracts (Cq1 and Cq3) on MRSA skin infection in the posterior upper backs of mice at the end of the experiment. (**B**) Efficacy of *Caralluma quadrangula* extracts on the bacterial load in murine model MRSA skin infection. Results are expressed as mean ± standard error. The dotted line represents the limit of detection of the viable count (log10 of 50 CFU = 1.7). * and *** indicate that the difference is significant at *p* < 0.05 and *p* < 0.0009, respectively (one-way ANOVA, Tukey’s post-hoc test).

Four groups of BALB/C mice, each group composed of eight mice, were injected with MRSA USA300 intradermally. An abscess at the site of infection was developed 24 h after infection. *C. quadrangula* extracts (Cq1 and Cq3) significantly decreased the bacterial load of MRSA USA300 in comparison with vehicle control and negative control groups (one-way ANOVA and Tukey’s test, *p* < 0.05) (Figure 3). MRSA USA300 count recovered from the Cq1-treated group was 1.724 and 1.777 logs lower than that of the vehicle control and negative control groups, respectively, while the bacterial count recovered from Cq3-treated group was 2.484 and 2.537 logs lower than that of the vehicle control and negative control groups, respectively. No significant difference was found between the MRSA USA300 count recovered from the vehicle control and the negative control groups (one-way ANOVA and Tukey’s test, *p* < 0.05) (Figure 3).

### 2.3. Extraction and Isolation of the Major Glycosides from N-Butanol Fraction

**Figure 4:**
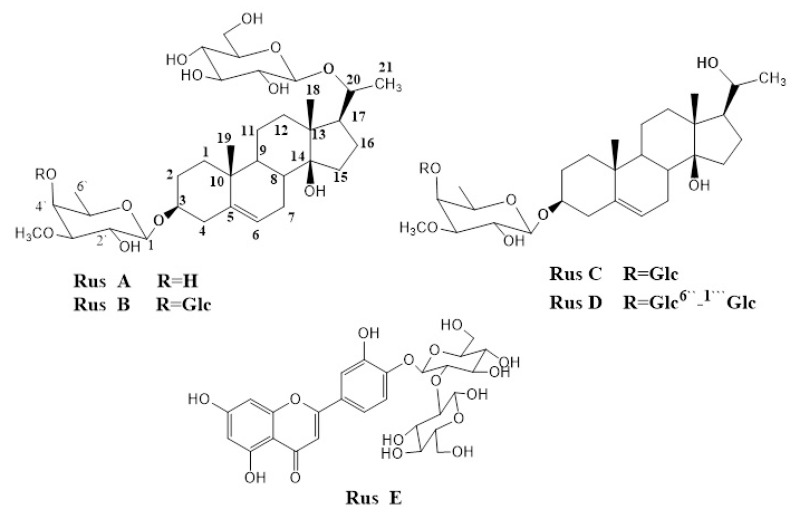
Chemical structures of russeliosides **A**–**E**.

Based on the results of the biofilm inhibition assay and the in vivo skin infection murine model, the *n*-butanol fraction was selected for further chromatographic separation for its bioactive components. Four major pregnane glycosides (**Rus A**–**D**) and one flavonoid (**Rus E**) were separated (Figure 4). The identification of the isolated compounds was achieved using ^1^H- and ^13^C-NMR and by comparison with authentic samples previously isolated from *C. russeliana* [28].

### 2.4. MBC and Antibiofilm Assays of the Isolated Compounds

**Figure 5:**
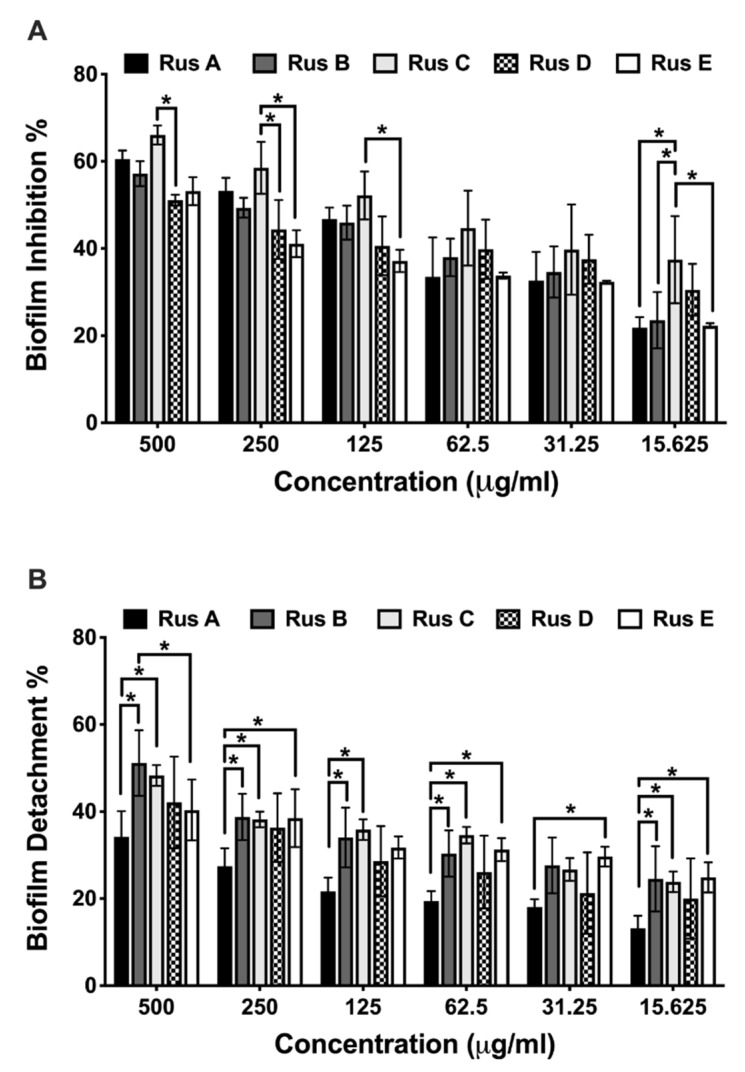
Antibiofilm inhibition activity. Effect of different concentrations (500–15.625 μg/mL) of the isolated *Caralluma quadrangular* compounds (**Rus A**–**E**) on MRSA USA300 biofilm. Results are expressed as mean biofilm inhibition % ± standard error (**A**) and mean biofilm detachment % ± standard error (**B**). * Indicates that the difference is significant at *p* < 0.05 (two-way ANOVA, Tukey’s post-hoc test).

Based on the promising activity of the tested *C. quadrangula* extracts against MRSA skin infection in the murine model, the isolated compounds (**Rus A**–**E**) from the active Cq3 fraction were tested for antibacterial and antivirulence (antibiofilm) activity against MRSA USA300 (Table 1). The five compounds had the same antibacterial activity against MRSA USA300 with MBC 5 mg/mL. The five isolated compounds significantly inhibited the biofilm formation of MRSA USA300 at different tested concentrations (500–15.625 μg/mL) (two-way ANOVA and Dunnett’s test, *p* < 0.0001) (Table 1). Compound **Rus C** showed the highest antibiofilm activity (IC_50_ = 0.139 mmole) among all the tested concentrations, followed by compound **Rus B** (IC_50_ = 0.279 mmole), and **Rus E** was the least with IC_50_ = 0.818 mmole (Figure 5A and Table 1). There was no significant difference in antibiofilm activity between compounds **Rus A** and **Rus D**, IC_50_ = 0.295 and 0.296, respectively (two-way ANOVA and Tukey’s test, *p* < 0.05). Compound **Rus C** showed significantly higher antibiofilm activity than compounds **Rus A**, **Rus B**, and **Rus E** at the least-tested concentration (15.625 μg/mL ≡ MBC/320) (two-way ANOVA and Tukey’s test, *p* < 0.05).

The five tested compounds significantly eradicated the previously formed MRSA USA300 biofilm at different tested concentrations (15.625–500 μg/mL, Figure 5B) (two-way ANOVA and Dunnett’s test, *p* < 0.0001). Interestingly, **Rus A** showed the least biofilm detachment activity at all the tested concentrations. **Rus B** and **Rus C** showed the highest biofilm detachment activity with no significant difference between them, with IC_50_ = 0.849 and 1.264 mmole, respectively (two-way ANOVA and Tukey’s test, *p* < 0.05).

## 3. Discussion

The World Health Organization (WHO) listed methicillin-resistant *S. aureus* (MRSA) and *A. baumannii* (MDRAB) as high-priority human pathogens. Being the primary causative agent of difficult-to-treat and persistent human infections, the ability of *S. aureus* and *A. baumannii* to make biofilm plays a crucial role in antibiotic resistance development [29]. *S. aureus* plays a notable role in skin infections to life-concerning invasive illnesses such as bloodstream infections, wound infections, and infective endocarditis [30]. The capability to form biofilm on both biotic and abiotic surfaces is a vital factor and a common cause of persistent infections related to indwelling medical devices and resistance to wide-spectrum antimicrobial agents. The inhibition of biofilm formation will inform the cells of bacteria against host immune clearance and antibacterial therapy and prevent the progression of infection [31]. Hence, targeting biofilm is an alternate protocol to contest bacterial infections.

Our work demonstrated the biofilm inhibitory efficacy of *C. quadrangula* extracts (Cq1, Cq2, and Cq3) against MDRAB AB5057 and MRSA USA300. Furthermore, the biofilm detachment activity of *C. quadrangula* extracts (Cq1 and Cq3) and their efficacy in a murine model of skin infection were also examined. *C. quadrangula* extracts were tested for their biofilm inhibition activity and biofilm detachment activity at very low concentrations below their MBC. The concentrations tested against MDRAB AB5057 were 1/20, 1/40, and 1/80 MBC, while the concentrations tested against MRSA USA300 were 1/40, 1/80, and 1/160 MBC. The results confirmed that the tested extracts exhibit significant antibiofilm activity against both multidrug-resistant strains without inhibiting cell growth, hence reducing the risk of the emergence of resistance against tested extracts and adopting an antivirulence approach in fighting resistant pathogens [32]. The inhibition of biofilm growth and development is more difficult to achieve than the detachment of preformed biofilm. These results were consistent with those found in previous studies [33,34].

*S. aureus* is a common cause of superficial skin infections [35]. The treatment of *S. aureus* infections has become complicated and more difficult due to the increasing incidence of multidrug-resistant *S. aureus* strains [36,37]. Therefore, the progress for discovering new compounds that are active as anti-multidrug-resistant strains, particularly against MRSA, is of great need [38]. *C. quadrangula* extracts had potent antibacterial activity against MRSA superficial skin infections in the mice model and enhanced the recovery of the induced skin infections. The extracts (Cq1 and Cq3) significantly decreased the bacterial load in the induced wound when compared to the control groups. These results indicate that C. *quadrangula* is a promising antibacterial candidate to be used in topical formulations against MRSA superficial skin infections. Our findings correlate with a recent study reporting significant antivirulence of *Illicium verum* Hook. Polar fraction against MRSA and MDRAB [12].

After the comparison of the biofilm inhibition activity of the isolated pregnane glycosides (IC_50_), we concluded that compounds with a lower number of sugar moieties at C-3 were more active (**Russ C** > **Rus D**). In the presence of two sugar moieties at C-3 (**Rus B** and **Rus C**), the absence of a sugar moiety at C-20 (**Rus C**) showed higher activity (**Russ C** > **Rus B**). Further future studies of more examples of pregnane glycosides are needed to find a sort of structure–activity relationship (SAR) among previously isolated pregnane glycosides.

Pregnane glycosides have been reported with interesting biological activities: antiparasitic, antimicrobial, antifungal, antisteroidogenic, anorectic, antioxidative, antiulcer, and antiarthritic [19,25,26,27,39]. From our study, we investigated their antibiofilm activity, where they showed significant activity. We also documented the structure–activity relationships of pregnane glycosides as antibiofilm.

## 4. Materials and Methods

### 4.1. Chemicals

Solvents used in this study were of analytical grades and purchased from El Gomhouria for Drugs Co. (Cairo, Egypt).

### 4.2. Plant Material

*C. quadrangula* (Forssk.) N.E.Br. (syn. *Monolluma quadrangula* (Forssk.) whole plant was collected from Al-Taif Governorate, Saudi Arabia, in February 2020. A specimen was left in the herbarium of Faculty of Pharmacy, Cairo University, Cairo, Egypt (No. 05.04.2020) and was authenticated by Dr. Emad Al-Sharif, Associate Professor of Plant Ecology, Department of Biology, Faculty of Science and Arts, Khulais, King Abdulaziz University, Saudi Arabia.

### 4.3. Extraction and Isolation of Major Compounds

The air-dried powder of *C. quadrangula* (300 g) was extracted with MeOH (3 × 1.5 L) using an Ultra-Turrax homogenizer to give on evaporation of the solvent 60 g of methanolic extract (Cq1). The methanolic extract (Cq1, 45 g) was partitioned consecutively with methylene chloride (4 × 250 mL), and *n*-butanol (4 × 250 mL) to get 6.5 of methylene chloride fraction (Cq2) and 33.2 g of *n*-butanol fraction (Cq3). Compounds 1–5 were isolated following the chromatography of Cq3 (20 g) on a silica gel column adopting the method reported by Al-Yahya et al. [28]. Their spectral data (^1^H- and ^13^CNMR) were compared with those reported in the reference paper [28]. The isolated compounds (**A**–**E**) were identified as russelioside **A** (**Rus A**), russelioside **B** (**Rus B**), russelioside **C** (**Rus C**), russelioside **D** (**Rus D**), and luteolin 4’-*O*-*β*-D-neohesperidoside (**Rus E**). The isolated compounds were first isolated from *C. russeliana* [28] and further isolated from *C. tuberculata* (unpublished data) and quantified by LC-MS [40].

### 4.4. Biological Assays

#### 4.4.1. In Vitro Studies

##### Bacterial Strains and Growth Conditions

We used the highly virulent MRSA (USA300) [41] and *A. baumannii* AB5057 [3,9] as the test organisms in this study. For biofilm assays, tryptic soy broth (TSB) and lysogeny broth (LB) were used for MRSA USA300 and multidrug-resistant *A. baumannii* AB5057 (MDRAB), respectively.

##### Minimum Bactericidal Concentration (MBC)

The minimum bactericidal concentration (MBC) was determined by the broth microdilution method [42]. One hundred microliters of double strength sterile Mueller–Hinton broth was pipetted into each well of a U-shaped-bottom sterile microplate, and the same volume of the tested extract or compound (100 mg/mL or 10 mg/mL, respectively) or DMSO (negative control) was added to the first well of each row. Serial dilutions (two-fold) were performed across the microplates (50–0.391 mg/mL for the extracts and 5–0.0391 mg/mL). Ten microliters of the bacterial inoculum (10^8^ CFU/mL) was then added to each well. The microplates were incubated for 24 h at 37 °C. All wells were spotted onto Mueller–Hinton plates and incubated for 24 h at 37 °C. The MBC was defined as the minimum concentration of the tested extract without detectable colonies of the target organism.

##### Antibiofilm Screening Assay

A static biofilm formation was assayed in a flat-bottom sterile ELISA plate as formerly reported [12]. Briefly, the bacterial suspension of MDRAB AB5057 or MRSA USA300 (10^8^ CFU/mL) was loaded in the plates (120 μL/well). The tested extract/compound (12 μL/well) was added to the bacterial suspension, and DMSO (solvent) was added instead of the tested extract/compound as the control. Each extract/compound was tested at concentrations below its MBC. The plates were incubated under static conditions. After the incubation period (24 h), cell growth was measured at 600 nm with a spectrophotometric plate reader (Biotek, Synergy 2, VT-USA). To quantify the established biofilm, each well was washed three times with saline (400 μL/well) then air-dried and stained with crystal violet (0.1% *w*/*v*, 150 μL/well) for 30 min. Then, the wells were rinsed three times with distilled water and left to dry completely. The crystal violet in the biofilm was solubilized by absolute ethanol (150 μL/well) for 20 min at 4 °C. The OD of the solubilized crystal violet solutions was measured by the plate reader spectrophotometer at 550 nm. For normalization, the reading was divided by OD_600 nm_ of the cell growth. The results were expressed as biofilm inhibition % using the following equation:

$$Biofilm\;inhbition\;\%=\frac{OD\;control-OD\;Test\;}{OD\;Control}\times 100$$

##### Biofilm Detachment Activity

*C. quadrangula* extracts (Cq1–3) and compounds (**Rus A**–**E**) were tested against previously established biofilm by MDRAB AB5057 or MRSA USA300. The assay was performed as previously reported [12]. The bacterial suspension was added to a flat-bottom ELISA plate (120 μL/well) and incubated under static conditions for 24 h at 37 °C. Cell growth was measured at 600 nm, then the wells were thoroughly emptied. The tested extract/compound was added (120 μL/well) at different concentrations. DMSO (solvent) was added instead of the tested extract/compound as untreated biofilm (reference value). Staining with crystal violet and measurements were applied as described above to quantify the remaining biofilm after the detachment activity of the tested extract/compound. The results were expressed as biofilm detachment % using the following equation:

$$Biofilm\;detachment\;\%=\frac{OD\;control-OD\;Test\;}{OD\;Control}\times 100$$

#### 4.4.2. In Vivo Study

##### Animals

Thirty-two BALB/C mice were supplied from a local supplier (The Modern Veterinary Office for Laboratory Animals, Cairo-Egypt). The animals (8 weeks old) were sheltered in plastic cages with a pellet diet and water (temperature: 25 ± 2 °C, humidity: 50 ± 10%, and light: 12/12 h light/dark cycle. The animal care and experiments were authorized by the Research Ethics Committee of the Faculty of Pharmacy, Cairo University (Approval No. MIC2669) and performed following the “Guide for the Care and Use of Laboratory Animals” published by the Institute of Laboratory Animal Research (Washington, DC, USA).

##### In Vivo Evaluation of *C. quadrangula* Extracts against MRSA Skin Infection in Mice

The murine model of MRSA skin infection was constructed as previously reported [12]. In brief, the upper backs of the mice were shaved and intradermally injected with 40 µL of mid logarithmically grown MRSA USA300 (5.5 × 10^8^ CFU) suspended in sterile buffered saline. Infected animals were randomly divided into four groups (n = 8/group). Forty-eight hours after infection, mice developed an abscess at the site of infection, which later developed into an open wound. Two groups were topically treated with *C. quadrangula* extracts (Cq1 and Cq3) at a concentration of 100 mg/mL (4 MBC). The third group was topically treated with the vehicle (25% *v*/*v* DMSO in water) and used as vehicle control. The fourth group was untreated and used as a negative control. The groups were topically treated by using 100 μL of the corresponding treatment once daily for three days at the site of infection. After 24 h from the last treatment, mice were euthanized, and the lesion was excised then homogenized in 0.5 mL saline (homogenizer, DAIHAN-scientific-pacificlab). Samples were diluted 10-fold and spotted on mannitol salt agar (MSA) for aerobic viable count. After incubation for 24 h at 37 °C, the colonies were counted, and the results of the treated groups were compared to those of the vehicle control and negative control groups.

## 5. Conclusions

*C. quadrangula* extract had a promising inhibition and detachment activity against biofilm formed by the highly virulent and multidrug-resistant *A. baumannii* AB5057 and MRSA USA300 with promising in vivo antibacterial activity against MRSA in a superficial skin infection in mice model. *C. quadrangula* is an effective antibiofilm agent and is a potential candidate against persistent infections of multidrug-resistant strains. These findings suggest that *C. quadrangular* may have a good potential use as an alternative defense against the rest of ESKAPE pathogens with further investigations needed to support this assumption.

## Figures and Tables

**Table 1:** IC50 of the five isolated *Caralluma quadrangula* compounds (**Rus A**–**E**) against MRSA USA300 biofilm.

	Biofilm Inhibition				Biofilm Detachment			
	LogIC50 ± SE	Mean IC50			LogIC50 ± SE	Mean IC50		
	-	(μg/mL)	(µmole)	(mmol)	-	(μg/mL)	(µmole)	(mmol)
**Rus A**	2.286 ± 0.083	193.2026	294.62±	0.295	3.62 ± 0.318	4164.101	6347.71	6.347
**Rus B**	2.358 ± 0.098	227.9783	278.7	0.279	2.842 ± 0.272	694.9902	849.62	0.849
**Rus C**	1.961 ± 0.141	91.43766	139.39	0.139	2.919 ± 0.125	829.7031	1264.79	1.264
**Rus D**	2.385 ± 0.122	242.6153	296.6	0.296	3.166 ± 0.496	1465.124	1791.1	1.791
**Rus E**	2.687 ± 0.093	486.1934	818.51	0.818	5.142 ± 0.943	138,566.6	233,277.1	233.277
